# *MtWRP1*, a Novel Fabacean Specific Gene, Regulates Root Nodulation and Plant Growth in *Medicago truncatula*

**DOI:** 10.3390/genes13020193

**Published:** 2022-01-22

**Authors:** Wei Chen, Yingjun Chi, Jinglong Zhang, Binqiang Bai, Xiaomin Ji, Yixin Shen

**Affiliations:** College of Agro-Grassland Science, Nanjing Agricultural University, Nanjing 210095, China; 2017220002@njau.edu.cn (W.C.); yingjunchi@njau.edu.cn (Y.C.); zjl_0416@163.com (J.Z.); binqiangbai@163.com (B.B.); xiaominji941219@163.com (X.J.)

**Keywords:** *MtWRP1*, N-terminal transmembrane domain, nodulation, plant growth, *Medicago truncatula*

## Abstract

Fabaceans symbiotically interact with nitrogen-fixing rhizobacteria to form root nodules. Some fabacean specific proteins play important roles in the symbiosis. WRKY-related Protein (WRP) is a novel fabacean specific protein, whose functions have not been well characterized. In this study, MtWRP1 was functionally characterized in *Medicago truncatula*. It contains a WRKY domain at C-terminal and a novel transmembrane (TM) domain at N-terminal, and its WRKY domain was highly similar to the N-terminal WRKY domain of the group I WRKY proteins. The TM domain was highly homologous to the eukaryotic cytochrome b561 (Cytb561) proteins from birds. Subcellular localization revealed that MtWRP1 was targeted to the Golgi apparatus through the novel TM domain. *MtWRP1* was highly expressed in roots and nodules, suggesting its possible roles in the regulation of root growth and nodulation. Both *MtWRP1*-overexpression transgenic *M. truncatula* and *MtWRP1* mutants showed altered root nodulation and plant growth performance. Specifically, the formation of root nodules was significantly reduced in the absence of *MtWRP1*. These results demonstrated that *MtWRP1* plays critical roles in root nodulation and plant growth.

## 1. Introduction

Fabacean (legume) is an important flowering plant family, containing the essential bean crops (e.g., soybean, *Glycine max*) and forages (e.g., alfalfa, *Medicago sativa*). Fabaceans could establish symbiotic interactions with rhizobia, which can facilitate nitrogen absorption of plants [1,2]. Biological nitrogen fixation is an efficient source of nitrogen [3,4]. The essential feature of the symbiotic nitrogen fixation system is the formation of inimitable plant root nodules. Nodule formation refers to two closely coordinated processes, infection, and nodule organogenesis [5,6,7]. These processes are governed by a number of unique signaling cascades, involving flavonoids and Nod factors, Type III, IV, and VI secretion system effectors, bacterial surface polysaccharides, NCR peptides, phytohormones, CLE and CEP peptides, and small RNAs [8,9,10,11,12,13,14,15].

To date, genome sequence information of several fabacean species has become available and shed light on the diversity, evolution, and special traits of fabaceans. Fabaceans have novel genes that diverged greatly from their progenitors with new functions on fabacean-specific traits. These novel genes could have arisen from the duplication and rearrangement of ancestral genes followed by rapid diversification [16]. For example, an unequal recombination event occurred between nodule-specific genes *nodulin-25* and a calmodulin gene, giving rise to the first symbiosis-specific Calmodulin-like protein CaML. The CaML family genes were localized within the symbiosome space, expressed specifically in root nodules, and played roles in mediating signal transduction between the host plant and microbial symbiont [17]. The ENOD8 family, evolved from fusions of known protein domains with an N-terminal signal peptide, are unique secretory proteins targeted to symbiosomes and bacteroids [18]. In addition, the oxygen-carrying heme protein leghemoglobin, unique to nodules, is likely a specialized product of divergence from ancestral plant hemoglobins that may have been derived from a hemoglobin gene in the last common ancestor to plants and animals [19]. Leghemoglobins enable the endosymbiotic fixation of nitrogen in fabacean nodules by channeling oxygen for bacterial respiration while maintaining a micro-oxic environment to protect oxygen-sensitive nitrogenase [20]. In short, the identification and functional analysis of novel fabacean specific genes will provide new insights into symbiotic interaction between fabaceans and rhizobia.

WRKY-related Protein (WRP) is a fabaceans specific protein that has a similar but unique sequence structure to WRKY transcription factors [21]. Considering the unique and special traits of fabaceans including symbiotic nitrogen fixation, the novel WRP variants might be involved in symbiosis process [22]. One soybean WRP, GmWRP1, positively regulated the formation of nodules [23]. However, the function and regulation mechanism of WRP has not been further studied. To investigate the characterization of WRP1, we studied the function of *MtWRP1* of *M. truncatula*.

## 2. Materials and Methods

### 2.1. Plant Materials and Growth Conditions

Seeds of *M. truncatula* R108 and *Nicotiana benthamiana* (tobacoo) were received from the National Center for Soybean Improvement (Nanjing Agricultural University, China). Two *MtWRP1* mutant lines (NF12769 and NF13984) were obtained from *Tnt1* retrotransposon population maintained by the Noble Research Institute [24].

*M. truncatula* seeds were scarified by sandpaper and germinated in the dark for 2 days. The germinating seeds were then transferred to plastic pots filled with quartz sand, and watered every three days with nitrogen or nitrogen-free FM liquid medium, respectively. For the expression analysis of *MtWRP1*, leaves, stems, and roots were collected from 4-week-old plants. Flowers were sampled from 8-week-old plants. Pods and seeds were collected from 90-day-old plants. For the expression analysis of *MtWRP1* during nodulation, nodules were harvested at 15-, 20-, and 30-day post-inoculation (dpi) with *Sinorhizobium mliloti* 1021 from *M. truncatula*. *N. benthamiana* seeds were sown in plastic pots filled with soil and watered every three days. All plants were grown in the growth chamber with a photoperiod of 16 h light/8 h dark at 24/18 °C in Nanjing Agricultural University.

### 2.2. Isolation and Sequence Analysis of MtWRP1

*MtWRP1* sequences were obtained by searching the core sequence of *WRP* in *M. truncatula* genome version 4.0v1 from Phytozome (http://www.phytozome.net/, accessed on 10 October 2018). The full-length cDNA of *MtWRP1* was cloned to pFGC5941 plant vector (GenBank No. AY310901) by gene-specific primers (Table S1), and transformed into *Escherichia coli* strain DH5α (Qingke, Nanjing, China). Correct sequences were confirmed by DNA sequencing. The DNAMAN v6 software was used to analyze the amino acid sequence of MtWRP1 [25]. A Neighbor-joining (NJ) phylogenetic tree was constructed by the MEGA 5 software [26]. Default settings were used for system parameters. Branch support values were based on 1000 nonparametric bootstrap replicates. The website (http://nls-mapper.iab.keio.ac.jp/cgi-bin/NLS_Mapper_form.cgi, accessed on 8 November 2018) was used to predict the nuclear localization signals of MtWRP1. TMHMM 2.0 Server (http://www.cbs.dtu.dk/services/TMHMM/, accessed on 8 November 2018) was used to analyze the transmembrane structure of MtWRP1.

### 2.3. Subcellular Localization

The coding sequences of MtWRP1, the N-terminal TM domain, and the C-terminal WRKY domain were PCR-amplified using gene-specific primers and fused to the GFP behind the CaMV 35S promoter in a modified pCAMBIA1300 vector, which generated MtWRP1-GFP, MtWRP1-NTD-GFP, and MtWRP1-CTD-GFP recombinant constructs. MtWRP1-GFP and MtWRP1-NTD-GFP were co-infiltrated with the ST-mRFP Golgi marker into tobacco leaves [27]. The MtWRP1-CTD-GFP recombinant construct was co-infiltrated with nuclear localization marker into tobacco leaves [28]. Two days after infiltration, approximately 1–2 cm^2^ segments of leaf tissue within the infiltrated zone were excised and placed on a glass microscope slide with a cover slip [29]. Confocal laser microscopy (Zeiss LSM700, Oberkochen, Germany) was used for fluorescence observation with−488 nm for GFP and 610 nm filter for mRFP. The empty plasmid was used as a control. Primers were listed in Table S1.

### 2.4. Analysis of Gene Expression with Real-Time RT-PCR

According to the supplier’s instructions, total RNA was isolated from different organs and nodules of *M. truncatula* using the Trizol reagent (TianGen, Nanjing, China). Extracted RNA was used for cDNA synthesis by the ReverTran Ace^®^ qPCR RT kit (Vazyme, Nanjing, China). qRT-PCR was carried out using QuantStudio 5 (Thermo Fisher, Waltham, MA, USA). The enzyme was the SYBR^®^ Green Real-time PCR Master Mix (Vazyme, Nanjing, China). Based on the evaluation of the four different validation programs (RefFinder, geNorm, NormFinder, and BestKeeper), *MtActin2* was selected as an internal control, which was identified as the most stably expressed gene in the three tested reference genes (Figure S1) [30,31,32]. The delta-delta Ct method was used to calculate the relative gene expression [33]. There were three independent biological replicates. All primers were listed in Table S1.

### 2.5. Identification and Genotyping of MtWRP1 Mutants

The *Tnt1* FSTs of *MtWRP1* mutants were obtained from the *M. truncatula* mutant database (https://medicago-mutant.noble.org/mutant/, accessed on 15 November 2018) [24]. The gene-specific and *Tnt1*-specific primers (Table S1) were used to isolate the *MtWRP1* homozygous mutants by PCR-based genotyping as previously reported [34]. The primers of MtWRP1-R and Tnt1-F2 were used to identify the *Tnt1* insertion in *wrp1-1*. The primers of MtWRP1-F and Tnt1-F2 were used to identify the *Tnt1* insertion in *wrp1-2*. The primers of MtWRP1-F and MtWRP1-R were used to identify the full-length of *MtWRP1*.

### 2.6. Generation of Transgenic Plants

*MtWRP1* was cloned into the XhoI/XbaI sites of the pFGC5941 vector. The resultant pFGC5941-*MtWRP1* construct was introduced into *Agrobacterium tumefaciens* strain EHA105 [35]. *M. truncatula* R108 leaf explants were transformed with *A. tumefaciens* containing pFGC5941-*MtWRP1* via the in vitro transformation-regeneration method [36]. The bar gene with phosphinotricin resistance was used to select transgenic *M. truncatula*. The overexpression of *MtWRP1* in transgenic lines was confirmed by qRT-PCR.

### 2.7. Growth and Phenotyping of M. truncatula Plants

Seeds of *MtWRP1* mutants, over-expression transgenic lines and *M. truncatula* R108 were selected for germination in filter paper at dark. After 2 d seedlings were transferred to quartz sand with nitrogen-free FM liquid medium. When the first true leaf was fully spread, *S. mliloti* 1021 was suspended in nitrogen-free FM solution, which was used to inoculate the roots. Plants and nodules were harvested in 30 dpi. The morphology of roots was measured by the instrument of Epson expression 1680 (Seiko Epson, Nagano, Japan).

## 3. Results

### 3.1. Identification of MtWRP1, a WRKY-Related Protein from M. truncatula

**Figure 1:**
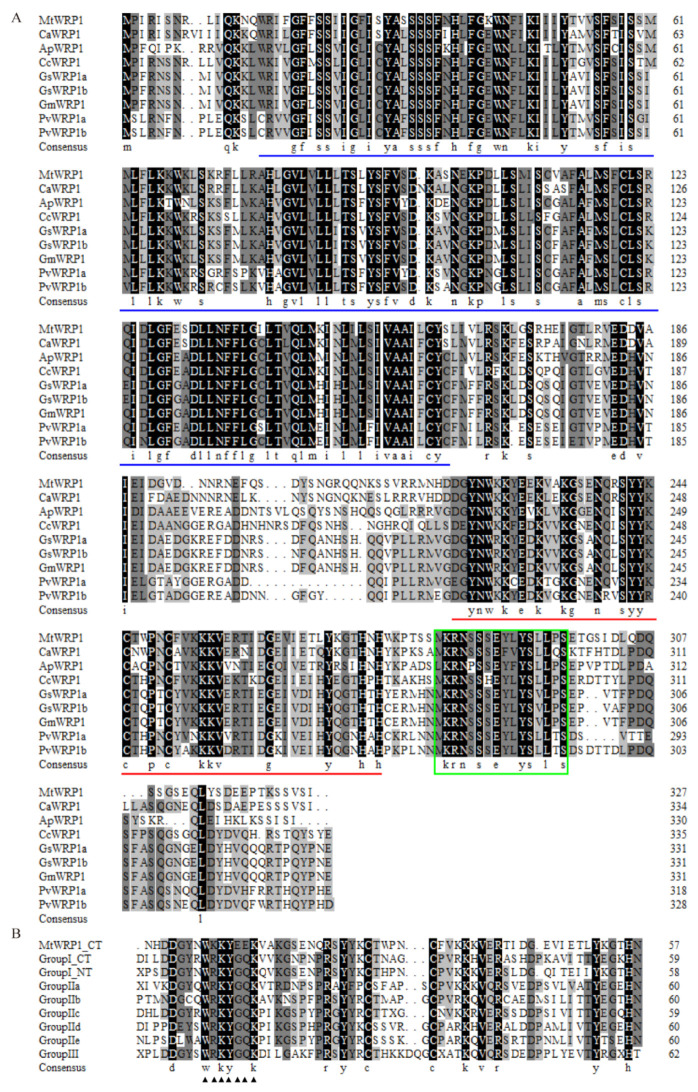
Multiple amino acid sequence alignment: (**A**) Close homologues of MtWRP1 in fabaceans. Amino acid sequences of the WRPs from *M. truncatula* (MtWRP1), *Cicer arietinum* (CaWRP1), *Abrus precatorius* (ApWRP1), *Cajanus cajan* (CcWRP1), *Glycine soja* (GsWRP1a and GsWRP1b), *Glycine max* (GmWRP1), and *Phaseolus vulgaris* (PvWRP1a and PvWRP1b). The TM domains of WRPs were indicated by blue lines. The WRKY domain of WRPs were indicated by red lines. The putative localization signals were indicated by the green box. (**B**) Alignment of the MtWRP1 WRKY domain sequence with the consensus sequences of the WRKY domains of seven WRKY subfamilies, including the N-terminal (NT) and C-terminal (CT) WRKY domains of group I WRKY proteins. The core sequences of the WRKY domain were indicated by triangles.

The full-length of *MtWRP1* (Medtr5g074200) contained an open reading frame of 984 bp that encoded a predicted protein of 327 amino acid residues with an estimated molecular mass of 37.5 kD and a calculated pI of 9.35. Using BLASTP (http://www.ncbi.nlm.nih.gov/BLASTp/, accessed on 8 November 2019), we identified one single ortholog of MtWRP1 in *Cicer arietinum, Abrus precatorius, Cajanus cajan,* and *Glycine max*, two orthologs in *Glycine soja* and *Phaseolus vulgaris* (Figure 1A), and no ortholog in non-fabacean plants. Multiple amino acid sequence alignment suggested that fabaceans’ WRP1 orthologs showed high sequence similarities.

**Figure 2:**
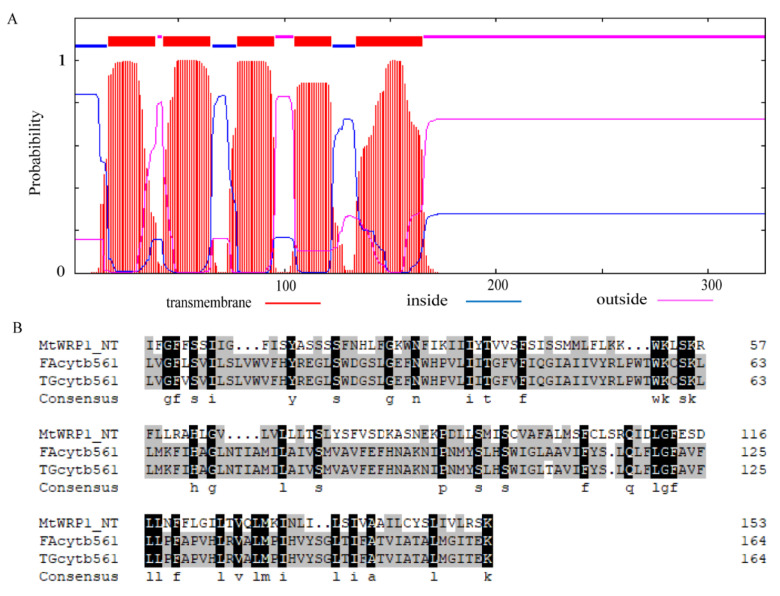
Structural analysis of TM domain at N-terminal MtWRP1: (**A**) Prediction of conserved TM domain in MtWRP1 as 5-passage transmembrane domain. (**B**) Sequence comparison of the TM domains of MtWRP1, FAcytb561 (XP_005049393.1), and TGcytb561 (XP_002198627.1).

Amino acid sequence analysis showed that MtWRP1 contained a WRKY domain at C-terminal and a novel TM domain at N-terminal (Figure 1A). Interestingly, the consensus sequences of WRKY domain in MtWRP1 was WKKYEEK; however, the great majority of WRKY protein was WRKYGQK (Figure 1B). Comparison with the seven groups of WRKY proteins showed that the WRKY domain of MtWRP1 shared 69.49% and 53.33% sequence identity with the N-terminal and C-terminal WRKY domains of group I proteins (Figure 1B), indicating highly similar to the group I WRKY proteins. However, unlike typical group I WRKY proteins with two WRKY domains, the C-terminal WRKY domain of group I WRKY proteins, which might be involved in the DNA-binding activity [37], was lacked in MtWRP1. A transmembrane prediction showed that the TM domain of MtWRP1 contained five-pass transmembrane helices (Figure 2A). BLAST search revealed that the TM domain was highly homologous to the eukaryotic cytochrome b561 (Cytb561) proteins of birds (Figure 2B).

**Figure 3:**
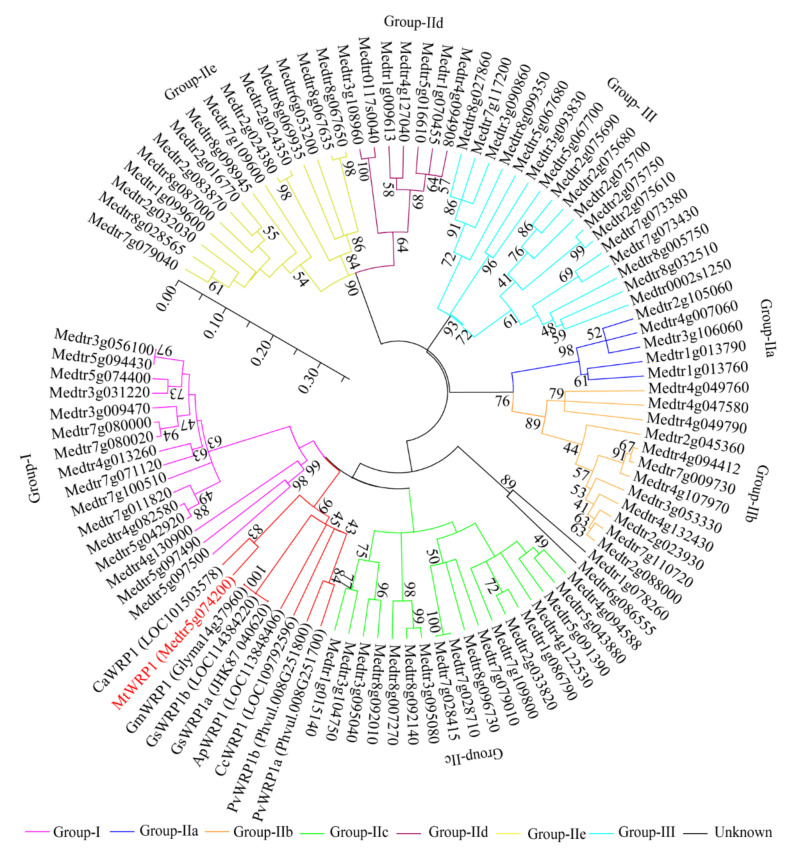
The phylogenetic analysis of MtWRP1, WRPs in different fabacean plants and the WRKYs in *M. truncatula*. The phylogenetic tree was generated from full-length amino acid sequences by MEGA 5 using the Neighbor-joining method. Numbers on branches indicated bootstrap values for 1000 replicates. The scale bar indicated the length of branch, reflecting the ancient divergence of these genes from each other.

Moreover, phylogenetic analysis was conducted to identify relationships between MtWRP1 and other proteins to reflect the ancient divergence [38]. Based on the results of Figure 1, eight WRP1s of different fabacean plants and all WRKYs of *M. truncatula* were selected to analyze the evolutionary relationships. Results corroborated that MtWRP1 was more closely related to CaWRP1 (Figure 3). In addition, it clustered with the group I WRKY proteins (Figure 3), suggesting that MtWRP1 might be evolved from the group I WRKYs. MtWRP1 had extensive modifications in the core sequence of WRKY domain (Figure 2B) that may lead to its novel biological functions.

### 3.2. Subcellular Localization of MtWRP1 to the Golgi Apparatus

**Figure 4:**
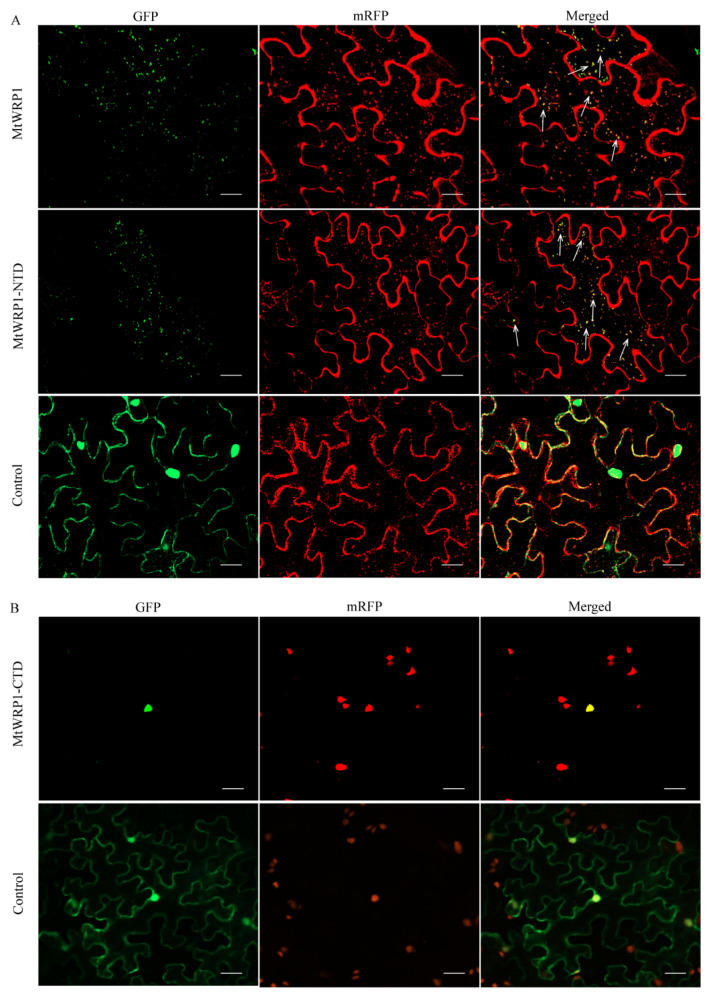
Subcellular localization: (**A**) Subcellular localization of MtWRP1 and TM domain at N-terminal of MtWRP1 (MtWRP1-NTD). A majority of MtWRP1-GFP and MtWRP1-NTD-GFP fluorescence signals were overlapped with mRFP Golgi marker signals. The arrows indicated the localization of GFP-fused proteins overlapped with the mRFP Golgi marker signals. Co-localization of free GFPs driven by CaMV 35S and mRFP Golgi marker were used as control. (**B**) Subcellular localization of WRKY domain at C-terminal of MtWRP1 (MtWRP1-CTD). MtWRP1-CTD-GFP fluorescence signals were overlapped with mRFP nucleus marker signals. Co-localization of free GFPs and mRFP nucleus marker were used as control. Bar, 20 µm.

Online prediction showed that MtWRP1 had potential nuclear and cytoplasm localization signal, suggesting that it was probably located in the nucleus and cytoplasm (Figure 1A). To further study the subcellular localization in plant cell, the MtWRP1-GFP fusion protein was transiently expressed in tobacco leaf epidermal cells. Confocal fluorescence imaging revealed that the green fluorescent signals were mostly overlapped with the mRFP Golgi apparatus marker, while the signals of free GFPs driven by CaMV 35S was distributed throughout the whole cell (Figure 4A). This result indicated that MtWRP1 was localized in the Golgi apparatus of tobacco epidermal cells.

MtWRP1 contained a novel TM domain and a WRKY domain. To verify which subdomain was significant for Golgi localization, the N-terminal domain of MtWRP1 was fused with GFP (MtWRP1-NTD-GFP) and co-expressed in tobacco leaves with mRFP Golgi apparatus marker. Meanwhile, the C-terminal domain of MtWRP1 fused with GFP (MtWRP1-CTD-GFP) was co-expressed in tobacco leaves with nuclear localization marker [28]. For MtWRP1-NTD-GFP, a majority of dispersed signals of green fluorescence were overlapped with mRFP Golgi apparatus marker (Figure 4A). For MtWRP1-CTD-GFP, all green fluorescences were overlapped with the nuclear signal (Figure 4B). These results indicated that MtWRP1 was targeted to the Golgi apparatus through the TM domain.

### 3.3. Analysis of MtWRP1 Expression

**Figure 5:**
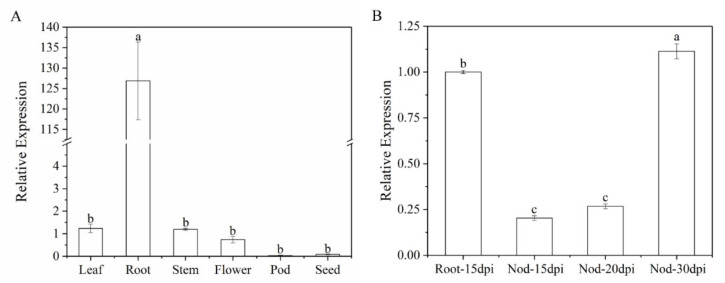
Expression of *MtWRP1* in *M. truncatula*: (**A**) The transcript levels of *MtWRP1* in different plant organs. Young leaves, stems, and roots were collected from 4-week-old plants. Flowers were sampled from 8-week-old plants. Pods at pod-bearing period and seeds at seed-filling period were collected from 90-day-old plants. (**B**) The transcript levels of *MtWRP1* in nodules (Nod 15, 20, and 30 dpi) inoculated with *S. mliloti* 1021. The gene expression in root at 15 dpi (Root-15 dpi) was used as control. Data represent mean ± SD (*n* = three independent biological replicates). Different letters above the standard error bars indicated a significant difference between the simples as determined by Duncan’s multiple range test (*p* < 0.05).

Furthermore, we analyzed gene expression patterns of *MtWRP1* in different organs. The result showed that the transcripts of *MtWRP1* were detected in a wide range of organs with different levels. The *MtWRP1* was significantly highly expressed in roots relative to other organs (Figure 5A). Meanwhile, the transcripts of *MtWRP1* accumulated during nodule development (Figure 5B). The unique expression patterns of *MtWRP1* suggested its possible roles in root growth and nodulation.

### 3.4. Functional Analysis of MtWRP1 in Transgenic M. truncatula and Tnt1 Mutants

**Figure 6:**
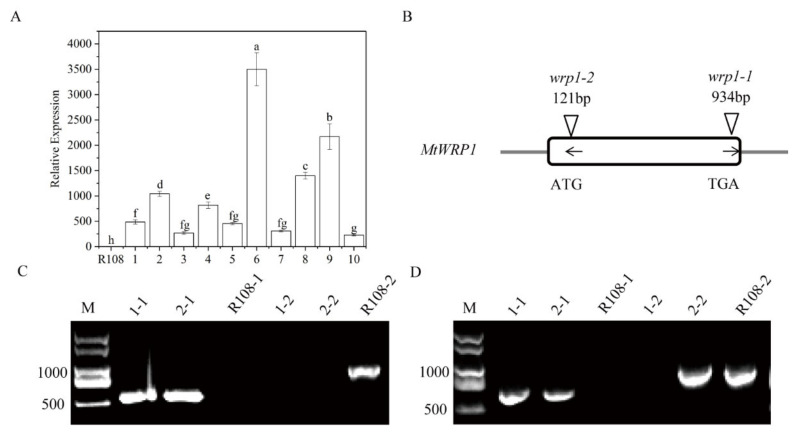
Identification of overexpression transgenic lines and *MtWRP1* mutants in *M. truncatula:* (**A**) The transcript levels of *MtWRP1* in transgenic *M. truncatula*. 1–10: transgenic lines. Data represent mean ± SD (*n* = three independent biological replicates). (**B**) Schematic representation of *MtWRP1* gene model and the *Tnt1* insertion sites. The box indicated the coding region of *MtWRP1,* which contained only one exon. The position of *Tnt1* insertions was indicated in base pairs (bp) with arrowheads. The orientation of *Tnt1* insertions was indicated by black arrows. (**C**) RT-PCR analysis of *MtWRP1* expression in *wrp1-1*. Lane 1-1, 2-1, and R108-1 were PCR-based identification of *Tnt1* insertion (544 bp) using primers MtWRP1-R and Tnt1-F2. Lane 1-2, 2-2 and R108-2 were PCR-based identification of MtWRP1 (984 bp) using primers MtWRP1-F and MtWRP1-R. R108 was used as the wild type control. M was a DL2000 DNA marker. (**D**) RT-PCR analysis of *MtWRP1* expression in *wrp1-2*. Lane 1-1, 2-1, and R108-1 were PCR-based identification of *Tnt1* insertion (677 bp) using primers MtWRP1-F and Tnt1-F2. Lane 1-2, 2-2, and R108-2 were PCR-based identification of MtWRP1 (984 bp) using primers MtWRP1-F and MtWRP1-R. R108 was control. M was a DL2000 DNA marker. Data represent mean ± SD (*n* = three independent biological replicates). Different letters above the standard error bars indicated a significant difference between the lines as determined by Duncan’s multiple range test (*p* < 0.05).

To further characterize its function, we generated *MtWRP1*-overexpression transgenic lines in *M. truncatula*. Ten transgenic lines were confirmed by qRT-PCR (Figure 6A). Three transgenic lines (OE1, OE2, and OE3) with different expression levels of *MtWRP1* were selected for further study.

We also identified two *MtWRP1* mutants (NF12769 and NF13984) in the *M. truncatula* mutant database of *Tnt1* FSTs [24]. After genotyping, homozygous plants of NF12769 and NF13984 were named *wrp1-1* and *wrp1-2*, with insertions in the exon region of *MtWRP1* at 934 bp and 121 bp downstream of the ATG start codon, respectively (Figure 6B). Expression levels of *MtWRP1* were determined by semiquantitative RT-PCR in *wrp1-1* and *wrp1-2* leaves. The results showed that the full-length *MtWRP1* transcripts were absent in *MtWRP1* mutants *wrp1-1 and wrp1-2*, whereas wild type R108 plants accumulated noticeable levels of *MtWRP1* transcripts (Figure 6C,D).

**Figure 7:**
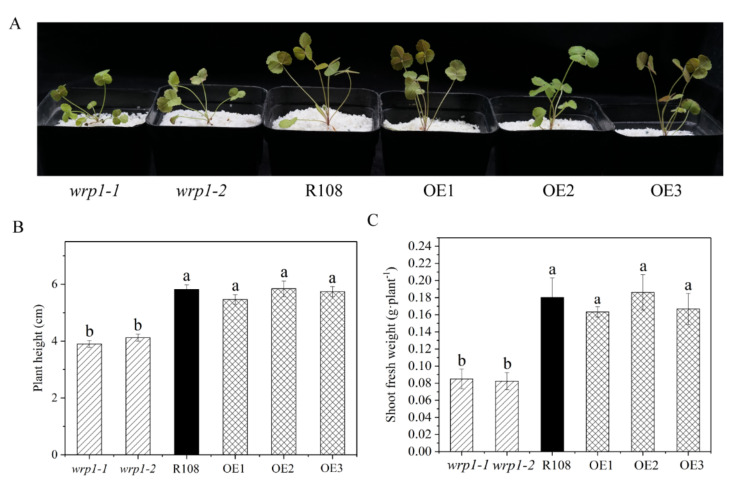
Effects of *MtWRP1* on plant growth and development in *M. truncatula*: (**A**) Growth phenotype of *MtWRP1* mutants (*wrp1-1* and *wrp1-2*), R108 and transgenic *M. truncatula* (OE1, OE2, and OE3) at 30 days post inoculation with *S. mliloti* 1021 under nitrogen-deficient conditions. (**B**) The plant heights of *MtWRP1* mutants, R108, and transgenic *M. truncatula*. (**C**) The shoot fresh weights of *MtWRP1* mutants, R108, and transgenic *M. truncatula*. Data represent mean ± SD (*n* = four independent biological replicates). Different letters above the standard error bars indicated a significant difference between the lines as determined by Duncan’s multiple range test (*p* < 0.05).

As shown in Figure 7, both *wrp1-1* and *wrp1-2* homozygous plants were significantly affected in their growth under nitrogen-deficient conditions, being much smaller and more compact than wild type R108 (Figure 7A). The *MtWRP1* mutants significantly decreased plant heights in comparison with wild type R108 (Figure 7B). The shoot fresh weights of *wrp1-1* and *wrp1-2* were significantly lower than that of R108 (Figure 7C). However, there was no significant difference between *MtWRP1*-overexpression transgenic *M. truncatula* and R108. Our results revealed that the lack of *MtWRP1* significantly affected the growth and development of *M. truncatula* significantly.

**Figure 8:**
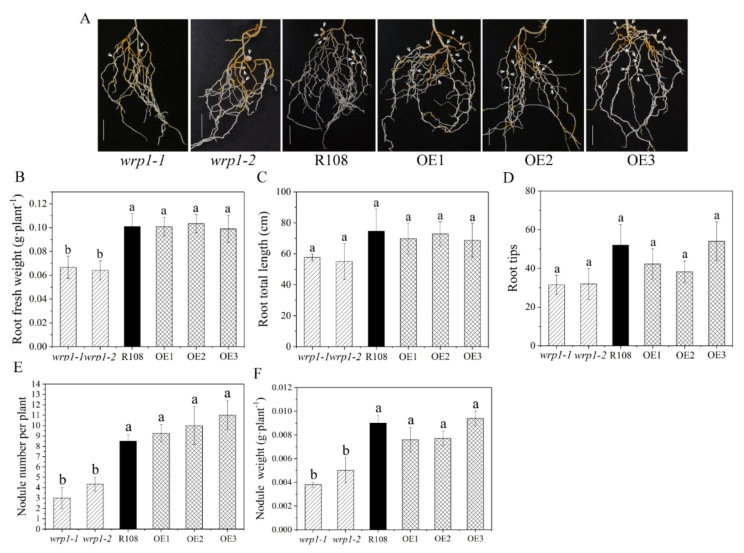
Effects of *MtWRP1* on root growth and nodulation in *M. truncatula:* (**A**) Nodulation phenotype of *MtWRP1* mutants (*wrp1-1* and *wrp1-2*), R108, and transgenic *M. truncatula* (OE1, OE2, and OE3) at 30 dpi under nitrogen-deficient conditions. Arrowheads indicated nodules. Bar, 1 cm. (**B**) The root fresh weights of *MtWRP1* mutants, R108, and transgenic *M. truncatula*. (**C**) The root total lengths of *MtWRP1* mutants, R108, and transgenic *M. truncatula*. (**D**) The root tips of *MtWRP1* mutants, R108 and transgenic *M. truncatula*. (**E**) The nodule number of *MtWRP1* mutants, R108, and transgenic *M. truncatula*. (**F**) The nodule weights of *MtWRP1* mutants, R108, and transgenic *M. truncatula*. Data represent mean ± SD (*n* = four independent biological replicates). Different letters above the standard error bars indicated a significant difference between the lines as determined by Duncan’s multiple range test (*p* < 0.05).

To elucidate whether *MtWRP1* played a role in root growth, nodulation, and/or rhizobial infection in *M. truncatula*, we compared the state of root growth and nodulation after inoculation with *S. mliloti* 1021 under nitrogen-deficient conditions among the mutants, R108, and OE plants. Compared with wild type R108, all the *MtWRP1*-overexpression lines, OE1, OE2, and OE3, had no significant alteration in root fresh weights, root total lengths, root tips, and nodule number as well as nodule weights (Figure 8). In addition, root total lengths and root tips showed no significant difference in the two *MtWRP1* mutants (Figure 8C,D). The root fresh weights of *wrp1-1* and *wrp1-2* were significantly lower than that of R108 (Figure 8B). Meanwhile, *wrp1-1* and *wrp1-2* exhibited reduced nodulation (Figure 8A), with nearly a 50% reduction in the number of nodules at 30 dpi (Figure 8E). The nodule weights were also significantly decreased in both *wrp1-1* and *wrp1-2* (Figure 8F). These results suggested that a mutation in the *MtWRP1* negatively affected nodulation under nitrogen-deficient conditions.

## 4. Discussion

### 4.1. Novel Structure of MtWRP1

In our study, a fabacean specific WRKY-related protein MtWRP1 was identified in *M. truncatula*. The WRKY domain of MtWRP1 was highly similar to the N-terminal WRKY domain of the group I WRKY proteins, suggesting that it might play roles in DNA-binding activity (Figure 1B). Group I WRKY proteins contain two WRKY domains, which play different roles in DNA-binding activities [37]. For instance, AtWRKY1 has two WRKY domains at C-terminal and N-terminal, respectively. The binding activity to W-box is mediated mainly through the C-terminal WRKY domain, while the effects of the N-terminal WRKY domain on the protein–DNA interaction is less [39]. Interestingly, the core sequence of the C-terminal WRKY domain in MtWRP1 was WKKYEEK (Figure 1A), exhibiting mutations at three amino acid residues in comparison with the conserved sequence WRKYGQK. The key residues mutations of MtWRP1 may affect the DNA-binding activity. It has been intensely discussed in recent years that the recognition and combination to W-box elements principally depends on the conserved WRKYGQK residues [40,41]. Particularly, the five consecutive residues RKYGQ are important for the DNA-bind activity of WRKY protein [42]. When the conserved WRKYGQK residues in the WRKY domain are substituted, the DNA-binding affinity will decrease. Meanwhile, any mutations of the conserved Cys and His of the zinc-binding motif abolish the protein–DNA interaction [43,44,45]. The inabilities of DNA-binding are caused by the point mutations on the correct structural scaffold. The residues of Lys, Gly, and Lys on WRKYGQK are the key residues for DNA-specific recognition and substitution, and each mutation of these residues resulted in non-specific binding [43]. GmWRP1, which has the key residues mutations in the C-terminal WRKY domain, loses the binding activity to the W-box [22].

TM domain of MtWRP1 existed only in fabaceans (Figure 1A), which implied that the novel TM domain appeared no earlier than 40–50 million years ago when the fabacean lineage appeared. This interpretation is consistent with the study that GmWRP1 and Exo70J genes with TM domain are distributed on chromosomes of soybean [22], which is an ancient tetraploid crop with two genome duplications that occurred approximately 59 and 13 million years ago [46]. Intriguingly, we searched the proteomes in plant and non-plant organisms, the result showed that the TM domain was highly homologous to cyt b561 proteins of two bird species, *Ficedula albicollis* and *Taeniopygia guttata* (Figure 2B). This feature reveals that the TM domain is structurally more similar to bird than plant cyt b561 proteins. Our study raises the intriguing possibility that the novel TM domain in fabaceans may have been derived from birds through horizontal gene transfer. Horizontal gene transfer describes the transmission of genetic material across species boundaries. It was reported that genetic exchange between rice (*Oryza sativa*) and fungi had occurred during their evolutionary history and added important metabolic traits to plant lineages [47]. Land plant *TAL* genes were derived from *Actinobacteria* through an ancient horizontal gene transfer event [48]. MtWRP1 probably underwent diversification through fusion with the TM sequence from cyt b561. Eukaryotic cyt b561 proteins with five to six α-helical TM segments are integral membrane proteins, which take part in ASC-mediated trans-membrane electron transport, supporting the regeneration of ASC [49,50]. Mammalian cyt b561 have also been suggested to function as iron reductases [51]. By homology, the novel TM domain of MtWRP1 may have the electron transport function.

### 4.2. Subcellular Localization of MtWRP1 to the Golgi Apparatus through TM Domain

MtWRP1 was targeted to the Golgi apparatus (Figure 4). Previous studies showed that WRKY TFs are generally located in the nucleus [52]. The possible reason could be that fusion of the WRKY domain with the novel TM domain led to the alteration of subcellular localization, which could generate new biological functions. Gene fusion is a process by which the complete or partial sequences of two or more distinct genes are fused into a single chimeric gene or transcript [16]. The subcellular localization will be changed in some cases after fusion. In soybean, GmWRP1 and Exo70J proteins, which have the domain fusion, alter the subcellular localization [22]. In flowering plants, the functional chimeric genes *coxll* and succinate dehydrogenase subunit SDH (*sdh3* and *sdh4*), which are formed by the fusions of mitochondrial genes and nuclear genes, are relocated to the nucleus [53,54]. The relocated proteins show adaptations to the physico-chemical properties of their altered cellular environments through the selective fixation of amino acid substitutions, which is followed by adaptive changes in the proteins’ functions [55,56,57,58].

Knowledge about the subcellular localization of proteins provides potentially significant information to unraveling their function. MtWRP1 was targeted to the Golgi apparatus, suggesting that the function of MtWRP1 might be related to well-known biomolecular processes in the secretory pathway of Golgi. The Golgi apparatus is the central sorting station of the eukaryotic secretory pathway, which is involved in the process of vesicle trafficking, endocytosis, exocytosis, autophagy, glycosylation, stress responses, and apoptosis [59,60,61,62,63,64,65,66,67,68]. In fabaceans, both the initiation and extension of infection threads (ITs), displaying polarized growth, require tip-directed vesicle trafficking, endocytosis, and exocytosis [69,70]. ITs grow toward differentiating symbiotic cells where they release rhizobia into symbiosomes through an endocytotic process. Within symbiosomes, the rhizobia grow and differentiate into nitrogen-fixing structures [7]. Studies demonstrate that vesicle trafficking, endocytosis, and exocytosis play essential roles in regulating fabacean–rhizobium symbiosis. MtWRP1 was located in the Golgi apparatus, which might take part in the colonization of ITs, the release of rhizobia from the ITs, and the formation of a nitrogen-fixing root nodule.

### 4.3. Biological Functions of Fabacean Specific MtWRP1

Nitrogen is an essential macronutrient for the growth of every organism. Plants have developed various strategies for acquiring nitrogen to adapt to a fluctuating nitrogen nutrient environment [71]. In fabaceans, when nitrogen availability from soil is low, root nodule symbiosis makes nitrogen from atmosphere available as a nutrient. However, symbiotic host plants consume photosynthetic products as an energy source for driving nodule development and nitrogen fixation. Unnecessary nodulation can be harmful, as plants lose carbon sources that could be used for their growth. Plants have a genetic mechanism to decrease nodulation if there are sufficient nitrogen sources available in their environment, saving the costs associated with nodulation. In the presence of high nitrate levels, the expressions of symbiotic genes are repressed, such as *cle-rs1* [72]. Moreover, nodulation was inhibited in *cle-rs1* plants to the same level as that in the wild type plants. Thus, there is a strong possibility that the function of *MtWRP1* on nodulation is important for *M. truncatula* growing under nitrogen-deficient conditions.

In our study, functional analysis through *MtWRP1*-overexpression transgenic *M. truncatula* and *MtWRP1* mutants revealed that the absence of *MtWRP1* led to significant reduction in nodule number and plant growth under nitrogen-deficient conditions. The *wrp1-1* and *wrp1-2* exhibited decreased plant heights and fresh weights, as well as reduced nodule number (Figure 7 and Figure 8 and Table S2). The reason could be that *MtWRP1* mutants could not provide sufficient nitrogen for plant growth. Meanwhile, the decline of photosynthesis and carbohydrate accumulation also gave feedback to roots and nodules for nitrogen fixation. Under nitrogen-deficient conditions, the nodule-fixed nitrogen is primarily transported to the aboveground part for shoot growth, and a tiny fraction is supplied to the roots and nodules growth. In soybean, up to 81.5%–87.1% of the nitrogen absorbed by the roots and fixed by the root nodules is supplied for shoot growth, leaving 12.9%–18.5% for roots and nodules growth [73]. In addition, the growth reduction in *MtWRP1* mutants may affect the photosynthesis biomass production, resulting in the reduction in nodules. Experimental evidence suggests that nodule functioning depends directly on current photosynthesis [74]. The limitation on photosynthesis may decrease carbohydrate supply to nodules, deplete carbohydrate concentrations in the nodules, and therefore constrain nodule activity and nitrogen fixation [75].

It is intriguing that there was no difference on nodule number and plant growth between OE lines and *M. truncatula* R108. The reason could be that MtWRP1 regulated the expression of target genes involved in the formation of root nodules and plant growth. Once MtWRP1 existed, the target genes would be activated or suppressed. The overexpression of MtWRP1 could result in a nonsignificant effect on nodule number and plant growth. However, when *MtWRP1* was silent, the nodule number and plant growth would be reduced. The data from these experiments suggest broad roles of *MtWRP1* not only in root nodulation, but also in the processes of plant growth and development.

## 5. Conclusions

In this study, MtWRP1 containing a WRKY domain at C-terminal and a novel TM domain at N-terminal was a fabacean specific WRKY-related Protein, which was targeted to the Golgi apparatus through the novel TM domain. *MtWRP1* positively regulated root nodulation and plant growth in *M. truncatula.* Plants have evolved multiple layers of regulation to balance nodulation and plant growth under any given environment intricately. What these additional components might be, and how they might interact with the MtWRP1-regulated processes, will be an interesting area for further studies.

## Data Availability

The data presented in this study are available in the article and Supplementary Materials.

## Supplementary Materials

The following supporting information can be downloaded at: https://www.mdpi.com/article/10.3390/genes13020193/s1, Figure S1: The validation of three reference genes for *M. truncatula*, Table S1: List of primers used in this study, Table S2: Effects of MtWRP1 on plant growth and nodulation.
