# Supplementary material for: MtWRP1, a Novel Fabacean Specific Gene, Regulates Root Nodulation and Plant Growth in Medicago truncatula
Source: Genes (Basel). 2022 Jan 22;13(2):193. doi: 10.3390/genes13020193 (PMC8871812; doi:10.3390/genes13020193)
Supplement: Supplementary file 1 [file genes-13-00193-s001.zip › genes-1445665-supplementary.pdf]

Figure S1. The validation of three reference genes for *M. truncatula*. The stability of reference genes in different organs was analyzed by RefFinder (A), geNorm (B), BestKeeper (C) and NormFinder (D). The stability of reference genes in nodules was analyzed by RefFinder (E), geNorm (F), BestKeeper (G) and NormFinder (H). The stability of reference genes in transgenic *M. truncatula* was analyzed by RefFinder (I), geNorm (J), BestKeeper (K) and NormFinder (L).

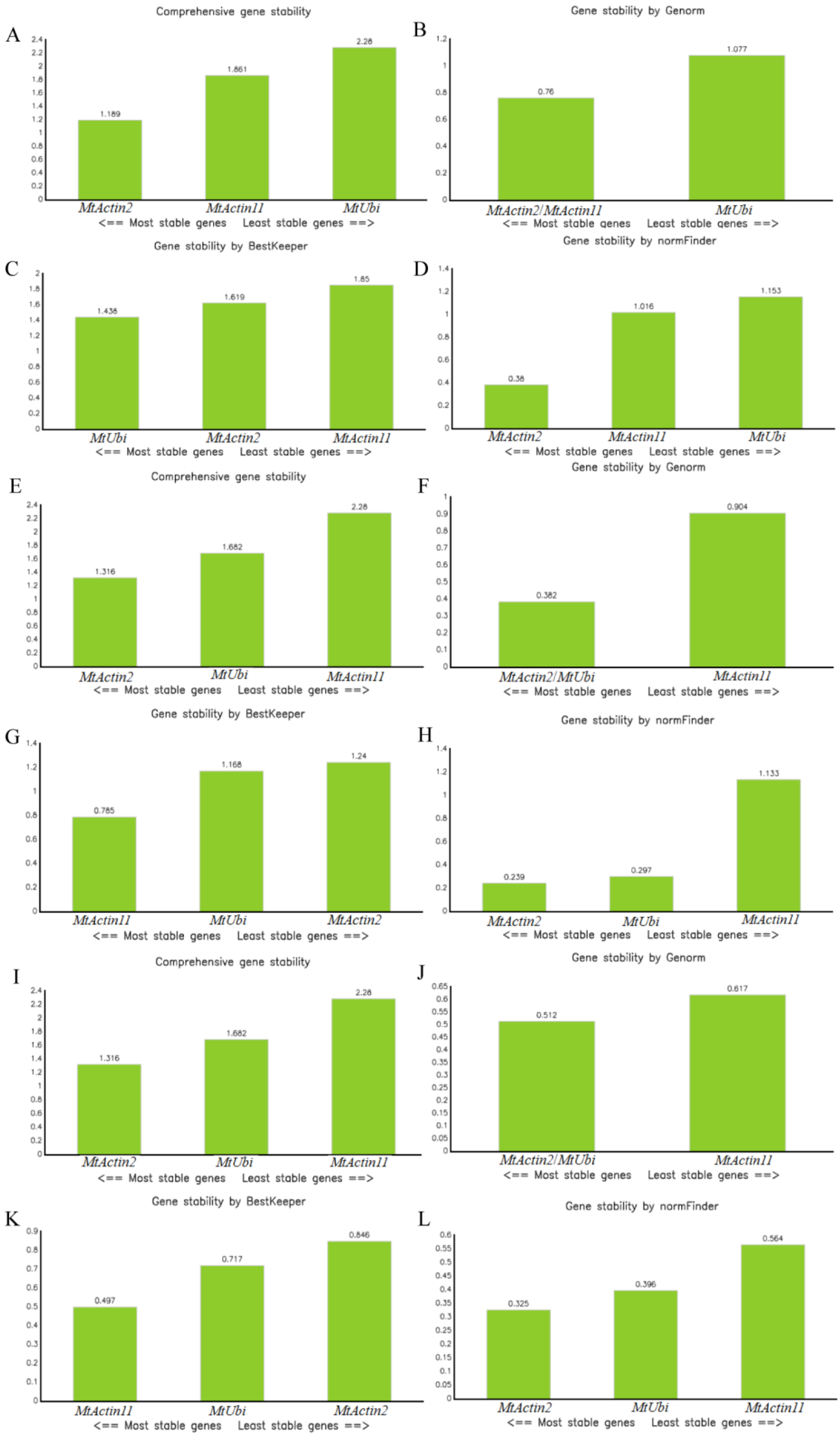
A
Comprehensive gene stability
1.189
1.861
2.28
MtActin2
MtActin11
MtUbi
<== Most stable genes Least stable genes ==>
B
Gene stability by Genorm
0.76
1.077
MtActin2/MtActin11
MtUbi
<== Most stable genes Least stable genes ==>
C
Gene stability by BestKeeper
1.438
1.619
1.85
MtUbi
MtActin2
MtActin11
<== Most stable genes Least stable genes ==>
D
Gene stability by normFinder
0.38
1.016
1.153
MtActin2
MtActin11
MtUbi
<== Most stable genes Least stable genes ==>
E
Comprehensive gene stability
1.316
1.682
2.28
MtActin2
MtUbi
MtActin11
<== Most stable genes Least stable genes ==>
F
Gene stability by Genorm
0.382
0.904
MtActin2/MtUbi
MtActin11
<== Most stable genes Least stable genes ==>
G
Gene stability by BestKeeper
0.785
1.168
1.24
MtActin11
MtUbi
MtActin2
<== Most stable genes Least stable genes ==>
H
Gene stability by normFinder
0.239
0.297
1.133
MtActin2
MtUbi
MtActin11
<== Most stable genes Least stable genes ==>
I
Comprehensive gene stability
1.316
1.682
2.28
MtActin2
MtUbi
MtActin11
<== Most stable genes Least stable genes ==>
J
Gene stability by Genorm
0.512
0.617
MtActin2/MtUbi
MtActin11
<== Most stable genes Least stable genes ==>
K
Gene stability by BestKeeper
0.497
0.717
0.846
MtActin11
MtUbi
MtActin2
<== Most stable genes Least stable genes ==>
L
Gene stability by normFinder
0.325
0.396
0.564
MtActin2
MtUbi
MtActin11
<== Most stable genes Least stable genes ==>

Table S1. List of primers used in this study.

Primer name	primer sequence (5′ -3′)	
MtWRP1-F	AGCCTCGAGATGCCCATAAGAATTTCC	Primers for *MtWRP1*
MtWRP1-R	AGCTCTAGATCATATCGATACCGAAGA	
MtWRP1-GFP-F	AGCCTCGAGATGCCCATAAGAATTTCC	Primers for generating GFP fusion constructs
MtWRP1-GFP-R	AGCGGATCCTATCGATACCGAAGA	
MtWRP1-NTD-GFP-F	AGCCTCGAGATGCCCATAAGAATTTCC	
MtWRP1-NTD-GFP-R	AGCGGATCCTTGAAATTCATTTCTATTGT	
MtWRP1-CTD-GFP-F	AGCCTCGAGATGGATTCTCAATCACAGATTG	
MtWRP1-CTD-GFP-R	AGCGGATCCTATCGATACCGAAGA	
MtWRP1-qRT-F	TGCAAGCAGCTCTTCTTTCAATCA	Primers for qRT-PCT
MtWRP1-qRT-R	ACACCCAAATGAGCTCTCAGCA	
MtActin2-qRT-F	TGGCATCACTCAGTACCTTTCAACTG	
MtActin2-qRT-R	ACCCAAAGCATCAAATAATAAGTCAACC	
Tnt1-F2	GTTGGATTGGTAGCCAACTTTGT	Primers for *MtWRP1* mutants

Table S2. Effects of MtWRP1 on plant growth and nodulation.

Lines	Plant height (cm)	Shoot fresh weight (g·plant^{-1})	Root fresh weight (g·plant^{-1})	Total length (cm)	Tips	Nodule number per plant	Nodule weight (g·plant^{-1})
wrp1-1	3.901±0.118^{b}	0.085±0.011^{b}	0.158±0.017^{a}	43.966±5.264	31.5±4.975	3.000±1.000^{b}	0.003±0.001^{b}
wrp1-2	4.120±0.124^{b}	0.082±0.010^{b}	0.139±0.018^{a}	42.344±1.953	32±8.010	4.333±0.667^{b}	0.004±0.001^{b}
R108	5.820±0.159^{a}	0.180±0.023^{a}	0.276±0.022^{b}	55.871±7.074	52±2.000	8.500±0.646^{a}	0.009±0.001^{a}
OE1	5.461±0.172^{a}	0.163±0.006^{a}	0.275±0.020^{b}	53.021±7.412	42.25±7.825	9.250±0.854^{a}	0.008±0.001^{a}
OE2	5.840±0.274^{a}	0.186±0.021^{a}	0.262±0.010^{b}	56.506±6.399	43.25±2.462	10.000±1.826^{a}	0.008±0.001^{a}
OE3	5.740±0.178^{a}	0.167±0.018^{a}	0.259±0.025^{b}	52.111±0.742	56.75±9.499	11.001±1.414^{a}	0.009±0.001^{a}
